# Exclusive breastfeeding practices and associated factors among lactating mothers of infants aged 6–24 months in the Kumasi Metropolis, Ghana

**DOI:** 10.1186/s13104-019-4723-0

**Published:** 2019-10-24

**Authors:** Joseph Yaw Yeboah, David Forkuor, Williams Agyemang-Duah

**Affiliations:** 1 Department of Geography and Rural Development, Kwame Nkrumah University of Science and Technology, Kumasi, Ghana; 2 Department of Planning, Kwame Nkrumah University of Science and Technology, Kumasi, Ghana

**Keywords:** Exclusive breastfeeding practices—lactating mothers, Kumasi metropolis

## Abstract

**Objective:**

In spite of the benefits associated with exclusive breastfeeding practice (EBP), the practice remains low in Ghana. This study investigates prevalence and factors associated with EBP among lactating mothers of infants aged 6–24 months in Metropolitan Kumasi. Cross-sectional hospital-based data were collected at 5 health facilities from 160 randomly sampled lactating mothers. Multivariate logistic regressions were performed to determine factors associated with EBP.

**Results:**

The prevalence of EBP was 50.6%. The study revealed that mothers aged 30–49 years (AOR = 1.948; 95% CI [1.146–3.310]), with normal delivery (AOR = 1.824; 95% CI [0.863–2.467]) and those who were unemployed (AOR = 1.202; 95% CI [0.557–2.593]) and without sore nipple (AOR = 1.890; 95% CI [1.534–3.484]) were significantly more likely to practise exclusive breastfeeding. The study further found that respondents with 3–4 deliveries were 0. 492 times significantly less likely to practise exclusive breastfeeding (AOR = 0.492; 95% CI [0.274–0.886]). The study has established the primacy of socio-demographic and health-related factors such as mothers’ age, employment status, number of deliveries (parity), mode of delivery and sore nipples in explaining EBP among lactating mothers. We recommend that policy on exclusive breastfeeding should consider multiple socio-demographic and health-related factors, especially, those associated with EBP.

## Introduction

Exclusive breastfeeding [EB] is the practice of feeding breast milk only to infants, excluding water, other liquids, breast milk substitutes and solid foods [1]. EB is endorsed as the surest way of scaling down child mortality in developing countries [2, 3], due to the fact that breast milk is regarded as the best source of nutrition for an infant [4]. Therefore, attempts to improve breastfeeding practices among lactating mothers will increase the number of infants benefitting from exclusive breastfeeding [5].

Globally, about seventeen in fifty of infants less than 6 months are exclusively breastfed [6]. Like the global situation, exclusive breastfeeding practice [EBP] remains low in developing economies with a 33% prevalence rate in sub-Saharan Africa [7]. The 2014 Ghana Demographic and Health Survey estimated Ghana’s EBP to be 52% [8]. In the developed world, less educated women are less likely to initiate exclusive breastfeeding [9]. Also, in developing countries, marital age and awareness on benefits of EB are associated with EBP [10].

Despite the documented factors on predictors of EBP, little is known from the perspective of lactating mothers in urban areas in Ghana. Predictors of EBP differ from one location to the other [10, 11], calling for specific data [10]. Besides, most studies did not consider the influence of health-related factors on EBP. Research findings in this area will add to the scientific body of knowledge and understanding of EBP and how to plan and position health interventions among lactating mothers. Furthermore, findings from this study may help scale down infant diseases and deaths and contribute towards the realization of the United Nations health-related sustainable development goals. It is within these gaps that this study examined the prevalence of EBP and its associated factors among lactating mothers of infants aged 6–24 months in the Kumasi Metropolis of Ghana.

## Main text

### Methods

#### Study area and sampling

Out of the fourteen government health care facilities (hospitals and health centres) providing maternal and child health services in the Kumasi Metropolis, five were randomly selected for the study. These were the Kwame Nkrumah University of Science of Technology and Technology (KNUST) Hospital, Manhyia Hospital, Kumasi South Hospital, Komfo Anokye Teaching Hospital and South Suntreso Hospital representing Oforikrom, Manhyia, Asokwa, Bantama, and Nhyiaeso sub-metros respectively (as at the time of field survey, Oforikrom and Asokwa were sub-metros of Kumasi Metropolis).

A hospital-based cross-sectional design was adopted using a quantitative approach. In the light of this, the design was employed to describe the demographic, socio-economic and health-related factors that predict exclusive breastfeeding practices. We were expecting at least 12% of the mothers to practise exclusive breastfeeding. Using Lwanga and Lemeshows’ [12] formula for sample size estimation for health research with an alpha value of 0.05, a sample size of 162 mothers was obtained. To cater for the non-response rate, the sample size was approximated to 170. Using simple random sampling technique, 34 mothers each with infants aged 6–24 months were selected from five health centres to participate in the survey. The study participants were requested to pick pieces of papers folded with “True’ and ‘False’ written on it. Those who chose ‘True’ were selected for the survey until the sample size earmarked for lactating mothers in each health care facility was achieved. Pregnant mothers were, however, excluded from the study [13], because they complained of weakness; hence, could not respond to the survey questions.

#### Data collection instrument

Structured closed-ended questionnaires were used as the principal data collection tool. The questionnaire was prepared in English and translated into Twi (local dialect of the study participants) for the benefit of the respondents. The questionnaire included respondents’ socio-demographic and medical characteristics. Other questions included in the questionnaires were framed as: did you practise exclusive breastfeeding for your infants for the first 6 months of life? What are your sources of information on exclusive breastfeeding practice? The validity of the questionnaire was determined through pre-test, review of related literature for language clarity as well as consulting an expert in the field of exclusive breastfeeding to ensuring quality control. The pre-test was conducted at the Asokwa Children’s Hospital to inform necessary corrections. This was followed with the actual data collection exercise which was carried out between January and February 2017. The questionnaire administration was done by five trained research assistants; however, the first and second authors supervised the data collection process. The estimated burden time for each questionnaire was between 25 min and 30 min. Written informed consent was obtained from the study participants before data were collected.

#### Measurements

*Dependent variable* The dependent variable was exclusive breastfeeding practice measured as ‘practice = 1 or not practice = 0’ over the first 6 months of life.

*Independent variables* The independent variables were marital status (1 = married; 2 = single), child’s gender (1 = male, 2 = female), disability status (1 = Yes, 0 = No), non-communicable disease (1 = Yes, 0 = No), sore nipple (1 = Yes, 0 = No), past illness (1 = Yes, 0 = No), religion (1 = Christianity; 2 = Muslim; 3 = Traditional), employment status (1 = Employed, 2 = Unemployed), mother’s age (1 = below 29 years; 2 = 30–49 years), educational level (1 = No education, 2 = Basic education, 3 = secondary education, 4 = tertiary education), mode of delivery (1 = through surgical operation, 2 = normal delivery), number of deliveries (parity) (1 = 1–2, 2 = 3–4, 3 = 5 or more), child’s age (1 = 6–11 months, 2 = 12–17, 3 = 18–24 months).

#### Data analysis

Data were subjected to critical validation checks and analyzed using Statistical Package for Social Sciences (version 16). Descriptive statistics were conducted to describe the dependent and independent variables. Logistic regression analysis was performed to estimate the associations between independent variables (socio-demographic characteristics and health variables) and dependent variable (exclusive breastfeeding practices). The variables were entered into a model simultaneously. Odds ratios (ORs) with 95% CI were reported at a significant level of 0.05 or less.

### Results

#### Socio-demographic and health characteristics of respondents

Overall, 160 study participants responded to the questionnaire items yielding a response rate of 94.12%. The majority (60%) of the respondents were aged 30–49 years, married (88.8%) with a secondary level of education (41.9%) and professed Christian faith (81.3%). Furthermore, most of the respondents were employed (88.1%). Moreover, a huge number of respondents had a female child (56.9%) aged 12–17 months (53.1%). The majority (68.8%) of the respondents reported past illness (68.3%), 31.2% had a disability, 27.5% had a sore nipple and 90% had no non-communicable diseases (see Table 1).

**Table 1:** Socio-demographic and health characteristics of respondents

Variable	Category	n = 160	%
Age	Below 29 years	64	40.0
	30–49 years	96	60.0
Marital status	Married	142	88.8
	Single	18	11.2
Education	No education	9	5.6
	Basic level	45	28.1
	Secondary level	67	41.9
	Tertiary level	39	24.4
Religion	Christianity	130	81.3
	Muslim	27	16.9
	Traditionalist	3	1.9
Employment status	Employed	141	88.1
	Unemployed	19	11.9
Child’s gender	Male	69	43.1
	Female	91	56.9
Child’s age	6–11 months	45	28.1
	12–17 months	85	53.1
	18–24 months	30	18.8
Parity (number of deliveries)	1–2	72	45.0
	3–4	51	31.9
	5 or more	37	23.1
Mode of delivery	Through surgical operation	39	24.4
	Normal delivery	121	75.6
Income (GHS)	Less than 500 cedis	49	30.6
	500 cedis or more	111	69.4
Past illness (3 months)	Yes	110	68.8
	No	50	31.2
Disability status	Yes	33	20.6
	No	127	79.4
Sore nipple	Yes	44	27.5
	No	116	72.5
NCDs	Yes	16	10.0
	No	144	90.0

#### Prevalence of exclusive breastfeeding practices among lactating mothers

The study has shown that 50.6% of the respondents practised exclusive breastfeeding whereas 49.4% did not practise it (see Table 2). The study reported that the majority (75.3%) of the respondents sourced information on exclusive breastfeeding from healthcare providers.

**Table 2:** Prevalence of exclusive breastfeeding practices

Variable	Response	n	%
Did you practise exclusive breastfeeding for your child for the first 6 months of life	Yes	81	50.6
	No	79	49.4
Sources of information on exclusive breastfeeding (applied to only those who practised EB)	Friends	4	4.9
	Family	14	17.3
	Media	2	2.5
	Health care providers	61	75.3

#### Factors influencing exclusive breastfeeding practices among lactating mothers

The study revealed that being between 30 and 49 years significantly increases the odds of practising exclusive breastfeeding by 1.948 (AOR = 1.948; 95% CI [1.146–3.310]). The results showed that respondents who were unemployed were 1.202 times significantly more likely to practise exclusive breastfeeding (AOR = 1.202; 95% CI [0.557–2.593]). Respondents with 3–4 deliveries were 0. 492 times significantly less likely to practise exclusive breastfeeding (AOR = 0.492; 95% CI [0.274–0.886]). Also, having a normal delivery significantly increases the odds of exclusive breastfeeding practices by 1.881 (AOR = 1.824; 95% CI [0.863–2.467]) Respondents without sore nipple were 1.890 times significantly more likely to practise exclusive breastfeeding (AOR = 1.890; 95% CI [1.534–3.484]) (see Table 3).

**Table 3:** Factors associated with exclusive breastfeeding practices

Variable	Exp(B)	95% CI for EXP(B)		*P* value
		Lower	Upper	
Mother’s age (years)				
Below 29 years (ref.)	1.00			
30–49 years	*1.948*	*1.146*	*3.310*	*0.014*
Step 1				
Marital status				
Married (ref)	1.00			
Single	1.997	0.822	4.853	0.127
Education				
None (ref)	1.00			
Basic	0.441	0.129	1.509	0.192
Secondary	0.526	0.155	1.786	0.303
Tertiary	0.385	0.110	1.350	0.136
Religion				
Christianity (ref)	1.00			
Muslim	1.550	0.753	3.190	0.234
Traditionalist	0.93	0.520	1.75.	0.999
Employment status				
Employed (ref)	1.00			
Unemployed	*1.202*	*0.557*	*2.593*	*0.039*
Parity (number of delivery)				
1–2(ref)	1.00			
3–4	*0.492*	*0.274*	*0.886*	*0.018*
5 or more	1.268	0.662	2.431	0.474
Child’s gender				
Male (ref).	1.00			
Female	1.265	0.758	2.113	0.368
Child’s age				
6–11 months (ref)	1.00			
12–17 months	1.991	1.158	3.422	0.113
18–24 months	1.537	0.871	3.704	0.106
Mode of delivery				
Surgical operation (ref.)	1.00			
Normal delivery	*1.824*	*0.863*	*2.467*	*0.011*
Income (GHS)				
Less than 500 Cedis (ref)	1.00			
500 cedis or more	0.699	0.399	1.224	0.210
Past illness (3 months)	1.00			
Yes (ref)	1.00			
No	1.418	0.821	2.450	0.210
Disability status				
Yes (ref)	1.00			
No	0.938	0.508	1.732	0.838
Sore nipple				
Yes (ref)	*1.00*			
No	*1.890*	*1.534*	*3.483*	*0.002*
Non-communicable diseases				
Yes (ref)	1.00			
No	0.509	0.227	1.132	0.270

Italic values indicate significance of *P* value (*P* < 0.05)

### Discussion

This study examined the prevalence and predictors of EBP among lactating mothers in Metropolitan Kumasi. The study found that 50.6% of the respondents practised exclusive breastfeeding for the first 6 months of their infants’ life which is higher than what had been reported in United States (24.4%) [14], Puerto Rico (31%) [15], India (between 46.3 and 48.8%) [16], Northeastern Tanzania (24.1%) [10] and Peninsular Malaysia (43.1%) [11] but was below the prevalence rate in Northwest Ethiopia (60.8%) [17], Northeast Ethiopia (81.1%) [18] and Pakistan (54%) [19]. These disparities could be due to differences in socio-cultural factors and awareness creation. Similar to a previous study, this study found that information on EBP was mainly sourced from health care providers [20].

The study found that mothers’ age, employment status, parity, mode of delivery and sore nipple were associated with EBP. The study found that maternal age significantly influenced EBP which confirmed findings by Sholeye et al. [21] and Maonga et al. [10] but was contrary to other studies [22, 23]. Even though we found an association between employment status and EBP [11, 24, 25], mothers who were unemployed were significantly more likely to practise EBP. Compared with employed mothers, unemployed mothers may be less restricted with work demands and as such have a higher likelihood of practising exclusive breastfeeding. However, there may be other underlying factors that explain gaps in EBP between employed and unemployed mothers in the study area which may call for further studies to inform appropriate measures to ensure universal practice of exclusive breastfeeding among lactating mothers.

Like many other previous studies [26, 27], this study found that mode of delivery was associated with EBP and that mothers with normal delivery were significantly more likely to practise exclusive breastfeeding. This is because mothers who delivered through surgical operation may have more problems with latching, positioning and a lot of pain [28], and physiological reasons compared with those with normal delivery [26]. Thus, women who delivered through surgical operation may need more attention, assistance and encouragement to improve rates of exclusive breastfeeding at discharge [26]. In line with Palla and Kitsantas [29], we argue that mode of delivery should be considered when providing lactation services and information to mothers.

The study revealed that parity was associated with EBP. Significance of the findings was the fact that respondents with 3–4 deliveries were significantly less likely to practise exclusive breastfeeding compared with their counterparts with 1–2 deliveries. To this end, health actors in their quest to increase EBP among lactating mothers should target more of those with 3–4 deliveries since these groups were less likely to practise exclusive breastfeeding.

Interestingly, the study found that sore nipple was associated with EBP of which those without sore nipple were significantly more likely to practise exclusive breastfeeding. This implies that constant and frequent breast screening by health workers would be a useful measure for early detection and prevention of sore nipple among lactating mothers. Such health programmes would go a long way to reduce the incidence of sore nipple among lactating mothers in order to facilitate EBP.

The strength of the study is that it constitutes one of the few studies that have examined the prevalence and predictors of EBP among lactating mothers of infants aged 6 and 24 months in the Kumasi Metropolis, Ghana. The unique aspect of this study is that it elicited views from multi-ethnic groups and culturally diverse lactating mothers. This study is also essential in contributing to the realization of the United Nations Sustainable Development Goal 3 of ensuring healthy lives and promoting wellbeing for all at all ages by 2030.

### Conclusion

The prevalence of EBP in this study was far below the recommended United Nations International Children’s Emergency Fund (UNICEF) exclusive breastfeeding coverage of 90%. The study found that mothers’ age, employment status, parity, mode of delivery, and sore nipple were associated with EBP among lactating mothers with infants aged 6–24 in the study area. The findings suggest the need for health care actors such as the World Health Organization, Ministry of Health, Ghana Health Services as well as District, Municipal and Metropolitan Health Directorates in Ghana to consider socio-demographic and health-related factors such as mothers’ age, employment status, number of deliveries (parity), mode of delivery and sore nipple in the formulation of policies on EBP among breastfeeding mothers since these variables were associated with EBP.

## Limitations

The study elicited views from mothers so there is a high possibility of under-estimation or over-estimation on exclusive breastfeeding practices. Also, the study made use of a cross-sectional survey which did not allow causal inferences and associations to be made from the factors influencing EBP.

## Data Availability

The datasets used and/or analyzed during the current study are available from the corresponding author on reasonable request.

## Abbreviations

EB: exclusive breastfeeding
EBP: exclusive breastfeeding practice
KNUST: Kwame Nkrumah University of Science and Technology
UNICEF: United Nations International Children’s Emergency Fund
WHO: World Health Organization
